# The reciprocal relationships between Chinese children’s perception of interparental conflict, negative thinking, and depression symptoms: A cross-lagged study

**DOI:** 10.3389/fpsyg.2022.857878

**Published:** 2022-09-30

**Authors:** Meirong Yang, Zhaoyan Meng, Huan Qi, Xiangfei Duan, Libin Zhang

**Affiliations:** ^1^School of Psychology and Mental Health, North China University of Science and Technology, Tangshan, Hebei, China; ^2^School of Educational Science, Ludong University, Yantai, Shandong, China; ^3^Collaborative Innovation Center of Assessment Toward Basic Education Quality, Beijing Normal University, Beijing, China

**Keywords:** perceived interparental conflict, negative thinking, depression symptoms, transactional model, trait-state modeling

## Abstract

The present longitudinal study used the traditional cross-lagged panel model (CLPM) and autoregressive latent trajectory model with structured residuals (ALT-SR) to examine the relationships between perceived interparental conflict (IPC), negative thinking (NT), and depression symptoms in Chinese children. Changes in these three variables over time were also examined, as well as the trait and state aspects of the relationships between them. A sample of 516 third-grade primary students completed questionnaires about IPC, NT, and depression three times over a period of 1 year, at 6-month intervals. The CLPM findings indicated that, assuming that stability of each variable across time was controlled, Chinese children’s perception of IPC significantly affected their level of depression through the mediating path of NT. After taking trait factors into account, among all the significant autoregressive and cross-lagged paths originally found in the CLPM, only one third remained significant in the ALT-SR model. More specifically, the ALT-SR model, revealed a driving effect of children’s NT on perceived IPC and depression symptoms. The CLPM model although elucidated the interplay among three variables, the ALT-SR model showed little evidence of their interrelated growth across time. Taken together, these results indicate that children’s perceived IPC in the long term are a stable trait, with few state-level fluctuations, and is not a significant within-person predictor of subsequent children’s internalization problems. These perceptions appear to contribute more to children’s general psychological tendency than do changes over time. The research is the first to test the reciprocal relationships between Chinese children’s perceived IPC, NT, and depression symptoms. The findings demonstrate that previously proposed theories about the bidirectional relation between IPC and children’s social adjustment, to some extent, may reflect a correlation at a trait level. Put another way, it is IPC’s central tendency to be sensitive in the long term as a stable trait that is associated with their children’s general tendency to show well adjustment. The study contributes to our understanding of that extend previous results and have implications for complementary theoretical and practical interventions. The complementary techniques of CLPM and ALT-SR models offer different insights into children’s internalization problems, and hold promise for supporting the building of more comprehensive children’s developmental theories that acknowledge the interconnectedness of different domains of mental health.

## Introduction

World Health Organization [WHO] (2017) has defined depression as a widespread mood disorder characterized by low mood, lack of vitality, and reduced verbal communication. Depressive symptoms are the physical and mental discomforts caused by depressed mood, which are typically characterized by sadness, loss of interest, despair, and stress or anxiety, and can be accompanied by poor social development and even suicidal ideation and attempts (Tuithof et al., 2018), and depressive symptoms are more common in the normal population than depression neurosis (Theunissen et al., 2022). In one cross-sectional sample survey, 23.9% of Chinese children met the diagnostic criteria for depressive disorder (Wang et al., 2016), while another study reported a higher incidence of depressive symptoms in children, reaching 28.4% (Liu et al., 2020). Investigating depressive symptoms in childhood is crucial, because early onset depression has been identified as a risk factor for depressive symptoms in late adolescence (Luby et al., 2014). Children with depression are at increased risk for adverse psychosocial and developmental outcomes, which often persist throughout life (Essau et al., 2020). However, research on depression in children in China has focused on depressed mood, while clinical medicine has focused more on diagnosis of depressive neurosis than on undiagnosed individuals with depressive symptoms. Therefore, research into the mechanisms underlying and predictors of depressive symptoms in children will assist the development of early intervention initiatives for depression.

According to Diathesis–Stress Theory, depression is influenced by the interaction of external pressure and internal quality (Ma et al., 2018). Stress generally refers to major life events and adverse changes in life, and diathesis refers to the physiological and psychological characteristics of depression (Su et al., 2021). On the one hand, stress stimulates diathesis, which makes depression more likely; that is, stress affects depression through the intermediary of diathesis. On the other hand, diathesis regulates the influence of stress on depression, whereby the influence of stress on depression increases as levels of diathesis decrease (Fan et al., 2018). Studies investigating the determinants of children’s mental health have suggested that exposure to early life stressors is a major risk factor for depressive symptoms in childhood (Theunissen et al., 2022). For children, conflicts between parents are one of the most stressful events they will have experienced (Mastrotheodoros et al., 2020; Liu et al., 2021).

In family life, interparental relationship quality not only hinges on parents’ happiness, but is also an important cornerstone that influences the socialization process of children and is closely related to their development. One study has shown that interparental conflict (IPC) is a widespread social problem in China, with the incidence of physical conflict between spouses at about 34.8% and mental conflict at about 55.6% (Cui et al., 2012). Interparental relationships are not only shaped by the experience of conflict, but also greatly influenced by the way in which spouses resolve conflict. Conflict resolution styles can be classified as constructive (e.g., successful conflict resolution, positive interpretation of unresolved conflict) or destructive (e.g., physical violence, verbal aggression). Constructive conflict resolution not only has no negative impact on child development, but also may in fact have a positive impact (Harold and Sellers, 2018; van Eldik et al., 2020; Warmuth et al., 2020). Therefore, we studied the impact of destructive IPC on child development. Evidence has suggested that parental divorce might relieve children of the stress resulting from continued exposure to high levels of conflict (Smith-Etxeberria et al., 2020). Although the percentage of divorce among Chinese parents is increasing over time, in general, marriages remain stable, with more than 93% of parents not getting divorced in China (Wang et al., 2021). IPC is more than twice as likely to affect child development than is divorce (Grych and Fincham, 2001). When parents are in conflict, children are not silent witnesses, but, in their own way, process these events cognitively and react emotionally.

Interparental conflict refers to verbal arguments characterized by shouting, insults, threats, displays of anger, and hostility, as well as physical aggression between couples, such as shoving and hitting (Giallo et al., 2021). Differences and conflicts between parents are an inevitable part of family life, but frequent and intense conflicts between parents usually cause psychological distress in adolescents, and several cross-sectional and longitudinal studies have found that children exposed to IPC are at increased risk of mental and physical developmental health problems (Harold and Sellers, 2018; O’Hara et al., 2019; O’Hara et al., 2021), for example, are more prone to cause depression symptoms (Yap and Jorm, 2015; Cheung et al., 2020; Ren et al., 2020; Warmuth et al., 2020).

The Emotional Security Theory of children who are exposed to IPCs, which proposes that IPC can undermine children’s emotional security both directly and indirectly through other influences (Cummings and Davies, 2010; Cheung, 2020). According to Emotional Security Theory, IPC affects children’s emotional expression and emotion regulation, and children with low emotional security will experience negative emotions for a long time without being able to regulate them, and might even develop psychological behavioral problems (He and Yu, 2015). Family Systems Theory considers the family as a whole, and holds that conflict between any two family members also affects other family members (Cox and Paley, 2003). Previous reports have shown that nearly a quarter of children witnessed one or more parental conflicts in the past month (Cihan and Ünalan, 2020). As important members of the family, children are particularly vulnerable to conflict between family members (e.g., perceived conflict between parents). The underlying reason for this susceptibility is their rapidly developing sense of self, which leads to increased sensitivity and mood swings that are more extreme (Zhou et al., 2017). An important psychological diathesis of inducing depression is negative thinking (NT), and long-term NT can lead to a higher level of depression in children (Ayhan and Kavak Budak, 2021). Previous researchers have found that children’s depression is positively correlated with NT, which is the core index of depression symptoms and a significant predictor of depression (Pirbaglou et al., 2013). These studies indicate that children’s depression may be influenced by IPC and NT. According to the Family System Theory (Cox and Paley, 2003), the influence of IPC on children’s internalizing problem behavior changes over time (Madigan et al., 2017). Studies have shown that the influence of IPC on children’s development may have a “sleeper effect” or “cumulative effect” (Vu et al., 2016). Therefore, cross-sectional or short-term longitudinal studies are unable to examine the dynamic impact of IPC on children’s development, and may ignore the potential impact in this relationship. According to the Transactional Model (McDonald Culp, 2010; Sy et al., 2013), an individual’s development is the result of the continuous and dynamic interaction between themselves and their surroundings. While the environment affects the individuals, their characteristics and behaviors can adversely respond to the environment (Goemans et al., 2018). Based on the above theoretical model, we hypothesized that IPC, NT, and depression symptoms influence each other, and a change in any of these variables may in turn alter their interactions.

According to Social Support Theory, the quality of parental relationships in a family is a significant determinant of children’s mental health (Assari et al., 2021). Numerous studies have explored the relationship between IPC and children’s depression symptoms through direct observation, parental report, and self-report, and these have consistently shown that children’s depression symptoms are significantly associated with the negative aspects of the parental relationship, especially IPC (Sheeber et al., 2001; Rudolph et al., 2008). Children often perceive IPC as their fault, yet often feel remorse and helpless when they realize that conflicts are inevitable, which leads to persistent negative emotional reactions and internalizing problems such as depression (Shelton and Harold, 2008). However, there is no uniform conclusion as to whether there is an interaction between IPC and children’s depression symptoms (Stice et al., 2004), and studies have shown that adolescent depressive symptoms are associated with negative interparental relationships (e.g., conflict), but not causally (Young et al., 2005). Previous studies have also reported that behavioral problems in adolescents with depression are associated with the parental relationship (Armstrong and Costello, 2002; Wolff and Ollendick, 2006). One longitudinal study found that depressive symptoms and perceived IPC can predict each other (Brière et al., 2013). IPC may affect the development of depressive symptoms by internalizing negative self-views, and the interpersonal interaction pattern of children with depression may cause negative reactions in the surrounding environment and aggravate IPC. Over time, a vicious cycle may develop that contributes to the early maintenance and worsening of depression. When children perceive conflict between parents as unavoidable, which triggers a depressive response. As the children’s depression increases, this negatively affects the harmony and stability of the family. Confirmation of children’s negative perceptions of themselves and others may further lead to depressive symptoms.

Negative thinking refers to the negative conceptions, thoughts, and thinking awareness that emerge when individuals are in a negative environment; the emergence of NT is characterized by automaticity, non-randomness, and a persistent presence, and is closely associated with the environment of individuals (Buschmann et al., 2018). The Cognitive Model Theory hypothesizes that negative cognitions (including NT and beliefs) play an important role in the appearance and maintenance of emotional and behavioral problems (Philippi et al., 2018). Numerous interventions and treatments that target negative perceptions have been found to be effective in alleviating emotional and behavioral problems in children and adolescents (Trick et al., 2016). Among other things, underlying schemas and NT can influence mode of thinking and interpretation of situations, and most psychological problems are said to stem from interpreting self-experience in a negative and critical way (Young et al., 2014). One longitudinal study showed that persistent NT predicted elevated levels of depression symptoms and the maintenance and recurrence of depression neurosis (Watkins, 2015; Spinhoven et al., 2018). According to the Cognitive Content Uniqueness Hypothesis, different NT contents correspond to different problems. For example, depressed children have significant cognitive distortions and biases about themselves and the world around them, and may even selectively focus on negative stimuli in the environment (Everaert and Joormann, 2020). It has also been found that individuals with depression symptoms and depressive neurosis have highly repetitive NT (Raes, 2012; Hijne et al., 2020). A significant association between NT and depression symptoms has been found in childhood, adolescence, and early adulthood (Brown et al., 2016). In conclusion, an increased tendency to engage in NT is a risk factor for depression. Moreover, some scholars believe that NT responds to individuals’ frequent and recurring personality tendencies or mind habits in response to stress, and can be considered as a personality trait that stabilizes over time (Liu and Thompson, 2017).

Using the Stress-Depression Susceptibility Theoretical Model, one study found that NT was dynamically associated with stressors and depression over time (Watkins and Roberts, 2020). Namely, stressors (such as IPC) and depression may drive individuals to engage in more NT; this repetitive NT may in turn trigger higher stress perception and exacerbate depression severity. Thus, NT may not only be a response to stress perception, but may also elicit higher stress perceptions and in turn increase the severity of depression. Furthermore, one longitudinal study has shown that NT is associated with increased the severity of depressive symptoms and can predict increased levels of depression (Spinhoven et al., 2019), which in turn predicts increased NT (Whisman et al., 2020). However, no studies have formally examined whether there is an interaction of test stressors (e.g., IPC) and NT with depressive symptoms over time.

Previous researchers have found that parent-reported IPC may underestimate the actual impact of children’s exposure to IPC due to the different criteria used to judge IPC (frequency, intensity, etc.) (Kitzmann and Cohen, 2003). As a result, researchers in this field no longer rely entirely on parental reports. At the same time, researchers believe that children’s understanding of IPC plays a key role in determining the impact of IPC on children’s emotions and behaviors. Based on their own past experiences, children, as active cognitive subjects and problem solvers, can try their best to understand and cope with the pressure brought about by IPC, and improper attribution of the conflict and the pressure resulting from an inability to cope with IPC are the main reasons underlying children’s internalizing problem behaviors (Grych and Fincham, 1990). Therefore, understanding how IPC is perceived by children can better predict his or her psychological development.

Based on findings of previous studies and theoretical models, we predicted that NT and depression symptoms in Chinese children are associated with increased and decreased perceived IPC, respectively, and, in particular, that children’s perceived IPC and NT predicted more depressive symptoms. Although there is long-standing evidence that has linked IPC and NT with depressive symptoms in children, the complex underlying mechanisms are not well understood, and for two reasons. First, most research has focused on the role of IPC and NT on children’s externalizing problem behaviors, and few studies have investigated their interactions with internalizing problem behaviors (e.g., depression symptoms). Second, the previously detected links between IPC, NT, and depression symptoms may not be completely free of confounding factors that covary with both parents’ and children’s characteristics at the trait level. Notably, attributes of individuals that are relatively stable across situations (i.e., between-person) are known as traits, whereas attributes of individuals that are malleable in nature (i.e., within-person) are known as states.

To summarize, we conducted a cross-lagged analysis of perceived IPC, NT, and depression levels in third-grade primary school children in a province in northern China. This 1-year follow-up study, with measurements taken at 6-month intervals, aimed to reveal the interaction patterns between children’s perceived IPC and internalized problem behaviors. This allowed us to gain insights into the complex interaction mechanisms between children’ perceived IPC, NT, and depression, and the extent to which these transactional relationships occur at the personal traits or time-varying states levels.

## Materials and methods

### Participants

Using the convenient cluster sampling method, third-grade students from 15 classes of three primary schools in a province in northern China were selected for a 1-year follow-up study, with data collected three times at 6-month intervals. Data were collected in mid-December of the new school year (T1), in mid-June of the following year (T2), and in mid-December of the following year (T3). At T1, 603 students participated in the survey (372 boys and 231 girls, T1 Mage = 9.03 ± 0.62; T2 Mage = 9.54 ± 0.62; T3 Mage = 9.99 ± 0.62), and 516 students participated in all three surveys, equating to a total loss of 87 people and an attrition rate of 14.43%. In terms of the family structure, 523 participants were reported to be in nuclear family, 38 in single parent family, 24 in step-family, and 18 unreported. There was no significant difference in sex, perceived IPC, NT, and depression at T1 between the participants who completed all three surveys. This study was approved by Ethics Committee of North China University of Science and Technology. All participation in this study was voluntary and informed consent was obtained from parents in advance.

### Measures

#### Children’s perception of interparental conflict scale

The Children’s Perception of Interparental Conflict (CPIC) scale, compiled by Grych et al. (1992) and revised by Chi and Xin (2003), was used to measure perception of IPC. The scale has ideal psychometric indexes and is suitable for Chinese subjects. The scale has 40 items, which cover the two following factors: conflict characteristics and conflict evaluation. This study only used the conflict characteristic factors to measure IPC. The conflict characteristics include three dimensions of conflict frequency (six items), conflict intensity (seven items), and conflict resolution (six items), such as “My parents rarely quarrel.” Participants rated each item on a four-point Likert-type scale from one (absolutely true) to four (not at all true). Scores were averaged across items, whereby higher scores indicated a higher frequency and intensity of IPCs perceived by Chinese children, and worse conflict resolution. The CPIC scale has demonstrated good internal consistency in a Chinese children sample for 7–13 years, Cronbach α = 0.88, the split-half reliability coefficient = 0.87 (Chi and Xin, 2003). In the current study, the Cronbach’s α coefficient of the scale at T1, T2, and T3 was 0.950, 0.944, and 0.875, respectively. Confirmatory factor analysis showed that the construct validity of the CPIC scale from T1 to T3 was good, χ^2^/df ≤4.697 in the present study, comparative fit index (CFI) ≥0.941, Tucker-Lewis index (TLI) ≥0.910, root mean square error of approximation (RMSEA) ≤0.066, standardized root mean square residual (SRMR) ≤0.050.

#### Children’s automatic thinking scale

The Children’s Automatic Thinking Scale (CATS), compiled by Schniering and Rapee (2002), was and revised by Sun et al. (2017). The scale includes 40 items covering the four following dimensions: personal sense of failure, physical threat, interpersonal threat, and hostility. Participants rated each item on a 5-point Likert-type scale ranging from 0 (not at all) to 4 (a lot). A higher score indicated a more severe NT. The CATS has demonstrated good internal consistency and construct validity in a Chinese children sample for first to six grades, Cronbach α = 0.92, χ^2^/df = 3.35, GFI = 0.97, NFI = 0.96, RMR = 0.06 (Sun et al., 2017). In the current study, the Cronbach’s α coefficient of the scale at T1, T2, and T3 was 0.977, 0.984, and 0.976, respectively. Confirmatory factor analysis showed that the construct validity of CATS from T1 to T3 was good in the present study, χ^2^/df ≤5.258, CFI ≥0.914, TLI ≥0.905, RMSEA ≤0.068, SRMR ≤0.040.

#### The center for epidemiological studies depression scale

The Center for Epidemiological Studies Depression (CES-D) scale, compiled by Radloff (1977), was used in our survey (Sun et al., 2009). Data were obtained using the Chinese version of the CES-D, using which we measured the frequency of individuals’ current depression symptoms. The scale contains 20 items that cover the four following dimensions: depression, positive emotion, physical symptoms and activity retardation, and interpersonal relationships. Items are scored as 0 (not at all) to 3 (a lot) to indicate the frequency of symptoms in the past week. Higher scores indicate lower mood states and accompanied by poor social development, and even suicidal ideation and attempts. The CES-D scale has demonstrated good internal consistency and construct validity in a large Chinese adolescent’s sample, Cronbach α = 0.88, χ^2^(164) = 7601.97, CFI = 0.95, GFI = 0.93, RMSEA = 0.07 (Sun et al., 2009). In the current study, the Cronbach’s α coefficient of the scale at T1, T2, and T3 was 0.907, 0.899, and 0.778, respectively. Confirmatory factor analysis showed that the construct validity of the CES-D scale from T1 to T3 was good in the present study, χ^2^/df ≤3.603, CFI ≥0.931, TLI ≥0.916, RMSEA ≤0.059, SRMR ≤0.046.

#### Demographic variables

Participants reported their sex (1 = boy, 2 = girl), age, family structure (1 = nuclear family, 2 = single-parent family, 3 = step-family), and subjective socio-economic status (SES). Subjective SES was assessed using the following question: “How would you rank your family’s financial situation in the local area?”. This was rated on a five-point scale ranging from one (relatively low) to five (relatively high) at T1.

### Procedure

Before data collection, consent from teachers and participants’ parents was obtained through participants’ head teacher, to whom we then explained the purpose and requirements of the research. Three schools and 15 classes were invited to participate in the survey, and all classes agreed to participate. The head teacher and psychology graduate students implemented the tests, and the questionnaire was distributed in every class. Before the test, the purpose, significance, and requirements were explained to children, as well as how to fill out the questionnaire. We reassured children and their parents that data were confidential and would only be used for academic research. We asked participants to complete the questionnaire as truthfully, carefully, and fully as possible to ensure good data quality. Children were told that they could ask any questions during the test, and were asked to return the questionnaire after completing it.

### Statistical analysis

Harman’s single-factor test was used to check for common method variance, then SPSS version 25.0 (SPSS Inc., Chicago, IL, United States) was used to obtain descriptive statistics, implement independent samples *t*-tests to investigate the sex differences in IPC, NT and depression, and examine correlations among study variables. Mplus 8.3 was used to implement the autoregressive cross-lagged panel model (CLPM) and autoregressive latent trajectory models with structured residuals (ALT-SR) analysis to determine the effect of IPC on NT and depression symptoms. Missing data were estimated using the full-information maximum likelihood procedure. The model was assessed using a combination of indices and criteria, such as χ^2/^df <5, CFI >0.90, TLI >0.90, RMSEA <0.08, and SRMR <0.05. The mediating effect was measured using bootstrapping with 5,000 resamples and was statistically significant when the 95% confidence intervals (CIs) did not contain zero.

## Results

### Common method bias control and testing

Given that the CPIC scale, CATS, and CES-D scale are self-reported scales, there may be common method bias in this study, which was tested using the Harman single-factor test (Podsakoff et al., 2003). Exploratory factor analysis was conducted on all variables at T1 and the factor analysis extracted 14 factors with characteristic roots greater than one; the first factor explained 31.81% of the total variance, which was less than the critical cut-off point of 40%, indicating that the common method bias was negligible (Tang and Wen, 2020).

### Descriptive statistics

The means, standard deviations, and intercorrelations between variables are shown in Table 1. Perceived IPC was significantly positively correlated with both NT and depression symptoms at T1, T2, and T3 (*r* = 0.283–0.563, *p* < 0.01), which indicates that a more severe perception of IPC was associated with a more severe NT and depression. The results of independent samples *t*-tests indicate that significant sex-related differences were found in IPC at T2 and T3, and in NT at T1 and T2 (*t* = 1.987, 2.184, *p* < 0.05). There were no significant sex-related differences in IPC or depression at T1. Therefore, we included sex as a covariate in the cross-lagged model for statistical control.

**TABLE 1:** Bivariate correlations and descriptive for study variables.

Variables	1	2	3	4	5	6	7	8	9	10	11
1. Age	–										
2. SES	0.066	–									
3. T1 IPC	–0.019	–0.015	–								
4. T2 IPC	–0.003	–0.046	0.517**	–							
5. T3 IPC	–0.014	–0.013	0.474**	0.522**	–						
6. T1 NT	–0.044	0.012	0.513**	0.466**	0.478**	–					
7. T2 NT	0.014	–0.004	0.403**	0.519**	0.563**	0.543**	–				
8. T3 NT	0.022	0.000	0.389**	0.454**	0.549**	0.456**	0.565**	–			
9. T1 Dep	–0.051	0.046	0.487**	0.347**	0.360**	0.489**	0.345**	0.283**	–		
10. T2 Dep	–0.011	–0.026	0.375**	0.563**	0.374**	0.373**	0.482**	0.410**	0.387**	–	
11. T3 Dep	0.002	–0.007	0.320**	0.379**	0.512**	0.365**	0.420**	0.536**	0.319**	0.400**	–
M ± SD (The whole sample)	–	–	99.98 ± 27.58	95.66 ± 28.69	84.69 ± 25.8	37.28 ± 35.24	32.44 ± 36.98	25.98 ± 31.23	23.92 ± 11.79	22.45 ± 11.43	18.07 ± 10.25
M ± SD (Boys)	–	–	101.32 ± 26.55	97.44 ± 28.03	87.22 ± 25.63	39.4 ± 35.12	34.96 ± 37.83	27.37 ± 32.7	24.41 ± 11.36	22.57 ± 11.34	18.14 ± 9.88
M ± SD (Girls)	–	–	97.53 ± 29	92.63 ± 29.63	80.26 ± 25.35	33.54 ± 35.19	28.2 ± 35.33	23.29 ± 28.34	23.16 ± 12.41	22.22 ± 11.62	17.81 ± 10.77
*t*	–	–	1.607	2	3.249	1.987	2.184	1.563	1.137	0.33	0.352
*p*	–	–	0.109	0.046	0.001	0.047	0.029	0.119	0.256	0.741	0.725

IPC, Interparental conflict; NT, Negative thinking; Dep, Depression; M, Mean; SD, Standard Deviation.

**p < 0.01.

### Analysis of the traditional cross-lagged panel model

#### The cross-lagged panel model analysis of perceived interparental conflict and depression symptoms

**FIGURE 1:**
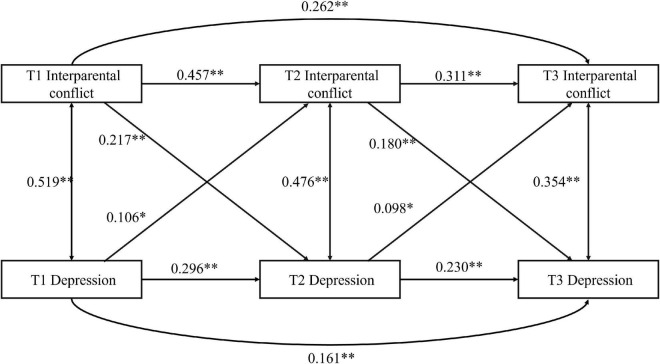
Cross-lagged model of Chinese children’s perception of interparental conflict and depression. The cross-lagged model controls for sex; The single-lined arrow line is the prediction relationship, and the double-lined arrow line is the correlation relationship, the solid line is the significant path, and the dotted line is the non-significant path; All path coefficients are standardized. The same below. *p < 0.05, ^**^p < 0.01.

Based on the correlation results, the CLPM was applied to explore the vertical relationship of perceived IPC and depression. The hypothetical model was constructed as shown in Figure 1, and the model yielded a good fit to the data, χ^2^_(2)_ = 3.70, *p* = 0.157, RMSEA = 0.039 (95% CI = [0.000,0.101]), CFI = 0.998, TLI = 0.964, SRMR = 0.010. The model path showed that, after controlling for the covariates of sex, age, subjective SES, family structure, autoregression and simultaneous correlation, perceived IPC at T1 positively predicted depression at T2 (β = 0.217, *p* < 0.001), depression at T2 positively predicted perceived IPC at T3 (β = 0.098, *p* = 0.037), depression at T1 positively predicted perceived IPC at T2 (β = 0.106, *p* = 0.023), and perceived IPC at T2 positively predicted depression at T3 (β = 0.180, *p* = 0.001). There was therefore an interactive relationship between perceived IPC and depression.

#### The cross-lagged panel model analysis of perceived interparental conflict, negative thinking, and depression symptoms

**FIGURE 2:**
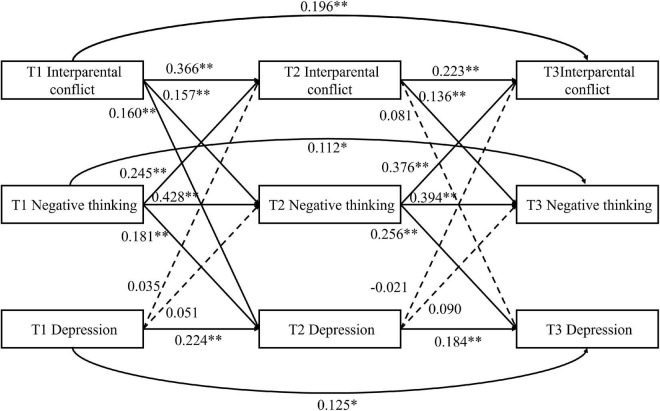
Cross-lagged model of Chinese children’s perception of interparental conflict, negative thinking (NT) and depression. *p < 0.05; ^**^p < 0.01.

To investigate the interaction between perceived IPC, NT, and depression, NT was added to the cross-lagged model shown in Figure 1 (see Figure 2). The model yielded a good fit to the data, χ^2^_(6)_ = 15.19, *p* = 0.019, RMSEA = 0.053 (95% CI = [0.020,0.086]), CFI = 0.995, TLI = 0.941, SRMR = 0.014.

The model path showed that, after controlling for the covariates of sex, age, subjective SES, family structure, autoregression and simultaneous correlation, perceived IPC at T1 positively predicted NT at T2 (β = 0.157, *p* = 0.001) and depression at T2 (β = 0.160, *p* = 0.005), perceived IPC at T2 positively predicted NT at T3 (β = 0.136, *p* = 0.005), but did not significantly predict depression at T3 (β = 0.081, *p* = 0.173), NT at T1 positively predicted depression at T2 (β = 0.181, *p* = 0.002), and perceived IPC at T2 (β = 0.245, *p* < 0.001), NT at T2 positively predicted depression at T3 (β = 0.256, *p* < 0.001) and perceived IPC at T3 (β = 0.376, *p* < 0.001), depression at T1 did not significantly predict NT (β = 0.051, *p* = 0.320) or perceived IPC at T2 (β = 0.035, *p* = 0.507), and depression at T2 did not significantly predict NT at T3 (β = 0.090, *p* = 0.106) or perceived IPC at T3 (β = −0.021, *p* = 0.684), and a suppressing effect of depression at T2 on IPC at T3 was observed.

The mediating effect of NT was measured using bootstrapping (5,000 resamples), which revealed a significantly indirect effect of perceived IPC at T1 on depression at T3 *via* NT at T2 (indirect effect = 0.040, 95% CI = [0.016, 0.073]). This indicated that NT plays a cross-temporal mediating role between perceived IPC and depression.

### Analysis of autoregressive latent trajectory model with structured residuals

**FIGURE 3:**
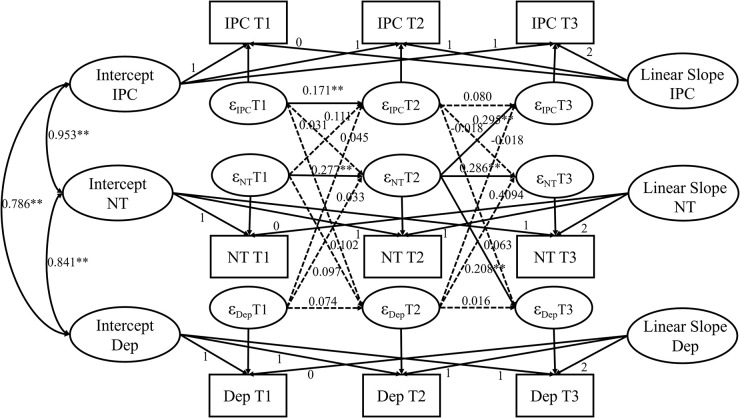
Autoregressive latent trajectory model with structured residuals (ALT-SR) for children’ perception of interparental conflict, negative thinking and depression symptoms. For simplicity, covariables (sex, age_T1_, and SES) correlations are not shown. IPC, interparental conflict; NT, negative thinking; Dep, depression symptoms; ^**^p < 0.01.

To disaggregate within- and between-person associations, we further explored the relationship between perceived IPC, NT, and depression using the ALT-SR model (see Figure 3; Berry and Willoughby, 2017). After controlling for the covariates of sex, age, subjective SES, and family structure, the ALT-SR model showed a good fit to the data that χ^2^_(3)_ = 4.84, *p* = 0.184, RMSEA = 0.033 (95% CI = [0.000, 0.084]), CFI = 0.999, TLI = 0.980, SRMR = 0.010.

In the model for children’s depressive symptoms, the correlations between traits of IPC and NT were significant (*r* = 0.953, *p* < 0.01). IPC and NT were significantly correlated with trait factors of depressive symptoms (IPC, *r* = 0.786, *p* < 0.01; NT, *r* = 0.841, *p* < 0.01).

In contrast to the CLPM models, not all the autoregressive paths in the ALT-SR models were significant. Although there were some state-level fluctuations in NT and perceived IPC (β_*s*_ = 0.171–0.286, *p*_*s*_ < 0.01), for perceived IPC, state changes only occurred between T1 and T2 (β = 0.171, *p* < 0.01). Moreover, among the six cross-lagged associations that were originally significant in the ARCL models (see Figures 1, 2), only 2 remained significant in the ALT-SR model (see Figure 3). Specifically, the state-level NT at T2 was significantly associated with both NT and perceived IPC at T3.

## Discussion

This longitudinal study sought to explore the complex and dynamic associations between parents and children. We compared results obtained from the CLPM and ALT-SR models to derive several important findings that could enhance our understanding of the association between perceived IPC, NT, and depression symptoms.

### The cross-lagged panel model results of Chinese children’s perceived interparental conflict, negative thinking, and depression symptoms

The CLPM results suggest that children’s perceived IPC significantly and stably affects children’s depression levels through the mediating pathway of NT, controlling for the stability of variables across time.

Specifically, first, this study showed that Chinese children’s perceived IPC positively predicted their depression, which is consistent with previous findings (Notten et al., 2017; Kopystynska et al., 2020). Namely, the stronger the perceived IPC, the higher the level of depression, which supports the Family System Theory. Our results highlight IPC as an important family factor that leads to Chinese children’s depression, which can seriously affect healthy development. The more severe the IPC, the more distant the parents’ relationship, the worse the family atmosphere, and the more psycho-emotional problems Chinese children have and the more likely they are to develop depressive symptoms (Brock and Kochanska, 2016). Our results also support Emotional Security Theory, which holds that IPC makes children feel the instability of their parents’ relationship, and the perceived IPC threatens their own and their family’s happiness; in turn, this aggravates the risk of psychological maladjustment (Fosco and Grych, 2008). From this perspective, our result provides a possible explanation for the impact of IPC on Chinese children’s depression.

Second, the intensity of Chinese children’s perceived IPC was significantly related to NT, which is consistent with previous results (Mason et al., 2009). We also found that a stronger perceived IPC was associated with a more serious the NT, and that NT can also significantly predict the level of perceived IPC. In addition, we found a significant correlation between NT and depression, which is consistent with existing work (Irfan and Zulkefly, 2021). This finding indicates that there is an interaction between depression and NT, whereby more NT is associated with a higher level of depression, and, in turn, the level of depression can also predict NT (Mazzer et al., 2019; Whisman et al., 2020). The present study supports the Cognitive Model Theory, in that it is possible that Chinese children’s perceived IPC, which is a negative life experience, gradually accumulated, resulted in negative beliefs, and influenced individuals’ attitudes and views about themselves, things around them, and even the future. With the accumulation of distorted, and extreme cognitions, these cognitions constituted NT. When children experience repeated and frequent NT, this in turn may drives them to form more negative cognitions, further influencing their perceptions of IPC, and creating a vicious cycle (Beck and Haigh, 2014; Young et al., 2014). The present study enhances our understanding of the mechanisms by which IPC influences NT in Chinese children. Importantly, this is the first study to systematically investigate the relationship between NT and perceived IPC over time. This new knowledge is important for validating theoretical predictions and identifying modifiable intervention strategies.

Third, the CLPM results also showed that a stronger perceived IPC resulting in more NT in Chinese children, and, in turn, higher levels of depression. This indicates that perceived IPC indirectly affects depression through NT. According to the Stress-Depression Susceptibility Theoretical Model, when perceptions of IPC increase, Chinese children’s tendency to engage in NT increases accordingly, and this higher NT in turn increases perceptions of IPC, which adds to depression. This suggests that the degree of stability and change in children’s tendency to adopt NT depends on their perception of environmental characteristics, such as the stressful environment of IPC and depression symptoms. Indeed, children’s tendency to engage in NT may be particularly elevated in the face of IPC and increased depression symptoms (Kingstona et al., 2014; Watkins and Nolen-Hoeksema, 2014). Interestingly, our study shown that children who are more inclined toward repetitive NT may in turn have elevated perceptions of IPC and depression levels. Thus, NT may not only be a response to IPC, but could also trigger higher levels of perceived IPC and increase the severity of depression symptoms. Future work could build upon our results to further investigate the mechanism underlying Chinese children’s depression. Our findings also support the hypothesis that Chinese children’s perception of IPC affects their level of depression by influencing NT.

### The autoregressive latent trajectory model with structured residuals results of Chinese children’s perceived interparental conflict, negative thinking and depression symptoms

The ALT-SR model allowed us to disentangle the trait (between-person) and state (within-person) aspects of perceived IPC, NT, and depression symptoms. Using this model, we analyzed the bidirectional relations between these variables. Unlike the CLPM findings, the ALT-SR models revealed fewer associations between perceived IPC, NT, and depression symptoms; fewer cross-lagged paths were significant compared with those in the CLPM. Nonetheless, the ALT-SR model results suggested that perceived IPC, NT, and depression symptoms have considerable trait-level stability and low state-level changes over time. Although less consistent than the CLPM model results, we did find some evidence for NT-driven effects, even when controlling for trait factors. Specifically, children’s NT was associated with future perceived IPC and depression symptoms.

Given that studies have shown that positive perceptions of IPC are associated with a prospective decline in depressive symptoms, it is a surprising pattern of findings that the ALT-SR results did not find that children’s internalized problem behaviors were influenced by IPC. These null findings do not necessarily indicate that IPC has no relevance to internalizing problem behaviors, and may simply indicate that state-level IPC changes may be more predictive of children’s internalizing behaviors. In addition, we found that children’s perceived IPC showed considerable stability over time and that these stable qualities are interrelated. Children’s perceived IPC may be an unstable state early on, but could gradually become less situationally reactive and a stable trait as the frequency of conflict increases and stabilizes, with fewer state-level fluctuations. In contrast, NT demonstrated substantial state fluctuations and changed over time. The increase in NT affected children’s enhanced perception of IPC and aggravated depression symptoms. Thus, children’s increased sensitivity to the perception of negative events may have led to greater perceived IPC and more severe depression symptoms. Children’s NT can be stressful for parents and may have caused even more IPC, thus leading to more perceived IPC by children.

Noteworthy is that the absence of bidirectional relationships does not mean that IPC is unimportant in child development research. Most existing theories about the bidirectional relationship between perceived IPC, NT, and depression may agree that parenting approaches to getting along are highly context-dependent and constantly changing (Troy et al., 2013; Haines et al., 2016), to some extent, reflect trait-level correlations. According to this interpretation, most of the CLPM model results may reflect trait-level covariations, rather than state changes over time. There have been ongoing debates about between- and within-person effects of the influence of parents on children within the family system (Nelemans et al., 2019). The consistent with previous findings that the influence of perceived IPC on children’s NT and depressive symptoms, as supported by CLPM models, were no longer supported when trait-level factors were taken into account (Hamaker et al., 2015). Known as a Simpson’s paradox (Kievit et al., 2013), if the between-person and within-person effects are not separated, the between-person correlation at the group level may oppose the within-person correlation at the individual level. It is these differences that remind us of the importance of considering differences between the trait and state level effects in future studies. Taking assessments of broader contexts (e.g., chronic stressors or periods of stress in life) into account may be important to better understand whether changes in habitual positive reappraisal are associated with increases or decreases in perceived stress (Everaert and Joormann, 2020).

That said, the CLPM findings do not necessarily mean that the traditional CLPM approach of modeling longitudinal data is outdated and of no use. Modeling the mean levels and aggregate effects in these models is better at capturing the chronicity and dose that may drive some causal impacts than methods that model within-person changes, such as the ALT-SR (Berry and Willoughby, 2017). However, many developmental theories are concerned with developmental changes or dynamic processes that happen within individuals, not at the population mean level (or between persons). Thus, we propose that, whenever possible, developmental researchers should consider using statistical tools that can separate within- and between-person effects when exploring relationships. These tools include, but are not limited to, the ALT-SR model.

### Implications

To our knowledge, the present study is the first to test the reciprocal relationships between Chinese children’s perceived IPC, NT, and depression symptoms. Our findings extend previous results, and have implications for complementary theoretical and practical interventions.

The interactions among three variables found in the present work are largely unsupported by existing research, and the transactional and reciprocal dynamics of family systems covered by many contemporary developmental theories should be revisited. For example, the Transactional Model of Child Development was originally developed to understand how individuals and their environment interact (McDonald Culp, 2010; Sy et al., 2013). However, it remains unclear to what extent the mutual interactions/influences at a given point in time, as proposed by these theories, are reflected in longitudinal data. The mechanisms underlying these changes (e.g., the length of time or dosage) are unknown. Thus, future research should examine this further.

That said, much like interventions studies that have separated intervention effects at the trait and state levels (Watts et al., 2017), or we need to find ways to help parents continually sustain the short-term gains in their parenting competence, such as interventions that have regular follow-ups.

### Limitations and future research

This study has some limitations that should be noted. First, it is difficult to avoid completely the social praise effect and common method deviation when using self-reported questionnaires to collect data. Also using a single self-reported questionnaires to assess IPC, NT and depression only from the child’s perspective may create a bias in the results. Future research could use third-party assessment methods, such as reported from a parental perspective, on-site observation or video scoring, to assess IPC, NT, and depression more effectively. Second, participants were third-grade students in a province in northern China, which limits the representativeness of the sample. Different samples could be tested to overcome this. Third, the follow-up duration was short, with only three follow-ups. Short-term longitudinal studies may overlook the potential impact of IPC on child development (Voelkle et al., 2012). Moreover, lagged effects may be positive in the short term and negative in the long term (Wagner et al., 2018). To overcome this, future work could increase the number and duration of follow-ups. Finally, there is no specific division of the different types of the impact of IPC on children’s development. The Marriage Balance Theory points out that the ratio of positive and negative interactions in marriage (such as the ratio of parental support to parental conflict) has an impact on children’s development (Katz and Gottman, 1993; Wang et al., 2021). In the future, researchers should further clarify the specific division of different types of IPC on children’s development, and comprehensively investigate the impact of positive interaction and IPC in marriage relations on children’s development.

## Conclusion

To conclude, the current study provides longitudinal evidence, on the one hand, the finding of CLPM indicated that under the premise that the stability of each variable across time is controlled, Chinese children’s perception of IPC significantly affected Chinese children’s depression level through the mediating path of NT. On the other hand, by distinguishing the trait and state factors of perceived IPC, in the ALT-SR model, the driving effect of children’s NT on perceived IPC and depression symptoms was observed. Taken together, it appears that children’s perceived IPC to be sensitive in the long term is a stable trait, and experiences less state-level fluctuations, and fluctuations in IPC are also important. These findings also highlight the importance of IPC on children’s development. These aforementioned findings probe us to think about what they mean for developmental theories and practical interventions. On the one hand, as the reciprocal effects between children’s perceived IPC and depression symptoms were largely unsupported in the current study, the transactional and reciprocal dynamics in the family system proposed by many contemporary developmental theories should be revisited. On the other hand, our results also suggest that interventions may only be valuable if researchers and practitioners work on psychological processes that can actually contribute to the trait-like changes in parents’ behaviors, such as focusing on their motivations and attitudes.

## Data availability statement

The raw data supporting the conclusions of this article will be made available by the authors, without undue reservation.

## Ethics statement

Approval was granted by the Medical Ethics Committee of North China University of Science and Technology (ethics approval number, 17009). Written informed consent to participate in this study was provided by the participants’ legal guardian/next of kin. Written informed consent was obtained from the minor(s)’ legal guardian/next of kin for the publication of any potentially identifiable images or data included in this article.

## Author contributions

MY prepared manuscript draft and contributed to supervision. ZM designed the study and wrote manuscript. HQ reviewed and revised manuscript. XD contributed to the conception and investigation. LZ collected and analyzed the data and contributed to review and editing, project administration, and supervision. All authors contributed to experimental design, discussing results, revision, and approval of the manuscript.
